# 

**Published:** 1905-10

**Authors:** 


### BOOKS RECEIVED.

The Diagnostics of Internal Medicine. By Glentworth Reeve Butler, M. D., Attending Physician to the Brooklyn Hospital. Octavo, pp. xxxiv-1168. With five colored plates and 288 illustrations and charts in the text. Second edition. New York and London: D. Appleton & Company. 1905. (Price, $6.00.)

Therapeutics: its Principles and Practice. By Horatio C. Wood, M.D., Professor of Materia Medica and Therapeutics in the University of Pennsylvania. Twelfth edition revised and adapted to the

The Principles and Practice of Medicine. By William Osler, M. D., Honorary Professor of Medicine, Johns Hopkins University, Baltimore. Sixth edition. Octavo, pp., 1161. New York and London: D. Appleton & Company. 1905.

A Manual of Clinical Chemistry, Microscopy and Bacteriology. By Ma Klopstock and A. Kowarsky, Berlin. Translated by Thew Wright, M.D. 12 mo, pp. 296. Illustrated. New York: Rebman Company. 1905.

Proceedings of the Medico-Psychological Association at the sixtieth annual meeting held at St. Louis, May 30-June 3, 1905.

Transactions of the Southern Surgical and Gynecological Association. Seventeenth session held at Birmingham, Ala., December 13, 14 and 15, 1905. W. D. Haggard, Secretary.

Progressive Medicine, Volume VII., September, 1905. A Quarterly Digest of Advances, Discoveries and Improvements in the Medical and Surgical Science. Edited by Hobart Amory Hare, M.D., Professor of Therapeutics and Materia Medica in the Jefferson Medical College of Philadelphia. Octavo, 298 pages. Philadelphia and New York: Lea Brothers & Company. (Per annum, in four clothbound volumes, $9.00; in paper binding, $6.00, carriage paid to any address.)

Lea's Series of Medical Epitomes. Edited by Victor C. Pendersen, M.D. Practice of Medicine. A Manual for Students and Prac titioners. By Hughes Dayton, M.D., Principal of the Class in Medicine, New York Hospital, Out-Patient Department. 12 mo., 324 pages. Lea Brothers & Co., Philadelphia, and New York. 1905 ($1.00, net.)

The Elements of Homeopathic Theory, Materia Medica, Practice and Pharmacy. Compiled and arranged from Homeopathic text-books by Drs. F. A. Boericke and E. P. Anshutz. 196 pages. Philadelphia: Boericke & Tafel. 1905. (Cloth, $1.00; postage, 5 cents.)

